# De Novo Assembly of a Sarcocarp Transcriptome Set Identifies AaMYB1 as a Regulator of Anthocyanin Biosynthesis in *Actinidia arguta* var. purpurea

**DOI:** 10.3390/ijms232012120

**Published:** 2022-10-11

**Authors:** Bei Niu, Qiaohong Li, Lijuan Fan, Xiaodong Shi, Yuan Liu, Qiguo Zhuang, Xiaobo Qin

**Affiliations:** 1 Key Laboratory of Coarse Cereal Processing, Ministry of Agriculture and Rural Affairs, Chengdu University, Chengdu 610106, China; 2 Sichuan Provincial Academy of Natural Resource Sciences, Chengdu 610015, China; 3 College of Life Sciences, Sichuan University, Chengdu 610064, China

**Keywords:** transcriptome, anthocyanin, regulation, MYB transcription factor, kiwifruit, *Actinidia arguta* var. purpurea

## Abstract

The kiwifruit (*Actinidia arguta* var. purpurea) produces oval shaped fruits containing a slightly green or mauve outer exocarp and a purple-flesh endocarp with rows of tiny black seeds. The flesh color of the fruit results from a range of anthocyanin compounds, and is an important trait for kiwifruit consumers. To elucidate the molecular mechanisms involved in anthocyanin biosynthesis of the sarcocarp during *A. arguta* fruit development, de novo assembly and transcriptomic profile analyses were performed. Based on significant Gene Ontology (GO) biological terms, differentially expressed genes were identified in flavonoid biosynthetic and metabolic processes, pigment biosynthesis, carbohydrate metabolic processes, and amino acid metabolic processes. The genes closely related to anthocyanin biosynthesis, such as phenylalanine ammonia-lyase (*PAL*), chalcone synthase (*CHS*), and anthocyanidin synthase (*ANS*), displayed significant up-regulation during fruit development according to the transcriptomic data, which was further confirmed by qRT-PCR. Meanwhile, a series of transcription factor genes were identified among the DEGs. Through a correlation analysis. AaMYB1 was found to be significantly correlated with key genes of anthocyanin biosynthesis, especially with *CHS*. Through a transient expression assay, AaMYB1 induced anthocyanin accumulation in tobacco leaves. These data provide an important basis for exploring the related mechanisms of sarcocarp anthocyanin biosynthesis in *A. arguta*. This study will provide a strong foundation for functional studies on *A. arguta* and will facilitate improved breeding of *A. arguta* fruit.

## 1. Introduction

Kiwifruit, belonging to the *Actinidia* family, is an important economic fruit crop beneficial for human nutrition, and is mainly produced in China, New Zealand, Italy, and so on [1]. One of the key traits of kiwifruits is their fruit colour, which is often determined by anthocyanins [2]. Nowadays, consumers are showing great interests in fruits with high levels of anthocyanins (red to purple pigments), because of their antioxidant and health-promoting properties [3]. Foods rich in flavonoids and anthocyanins are considered to play a crucial role in disease prevention [4]. *Actinidia arguta* var. purpurea fruits have various biological functionalities, such as antioxidation, cardioprotection, and anti-inflammation, which could be used for nutritional supplements.

Anthocyanins, which are water-soluble pigments that belong to the flavonoid family, are mainly located in cell vacuoles. Their color is affected by the intravacuolar environment, appearing as red, blue, and purple in various plant tissues such as flowers, fruits, seeds, and leaves [5]. Anthocyanins commonly found in plants mainly include cyanidin, delphinidin, pelargonidin, malvidin, and so on [6,7]. Some studies have characterized the biosynthetic pathways of anthocyanins and flavonoids and their functional importance in the development of other plant fruit [8,9,10]. Flavonoids are synthesized via the phenylpropanoid pathway by conversion of phenylalanine to 4-coumaroyl-CoA. Three different classes of flavonoids, including anthocyanins, are synthesized by chalcone synthase (*CHS*). Under the catalysis of leucoanthocyanidin dioxygenase (*LDOX*/*ANS*), leucoanthocyanins are converted to anthocyanindins, and are further glycosylated by uridine diphosphate glucose: flavonoid-O-glycosyltransferase (*UFGT*) to form cyanidin derivatives [11]. Meanwhile, genes encoding these enzymes in the flavonoid biosynthesis pathway are regulated by synergistic transcription, because these enzymes may also serve as metabolites, which will affect the total efficiency of this pathway [12,13].

In plants, the genes involving in the flavonoid biosynthetic pathway are largely regulated at the transcriptional level. So far, the expression of anthocyanin biosynthetic genes have been reported to be regulated by a complex of MYB transcription factors, basic helix–loop–helix (bHLH) transcription factors, and WD-repeat proteins (the MYB-bHLH-WD40 complex, MBW) [11,14]. MYB, including Arabidopsis MYB75 (AtPAP1) and AtMYB90 (PAP2) [15], petunia AN2 [16], grape MYBA1 and MYBA2 [17,18], sweet potato MYB1 [19], apple MYB10/MYB1/MYBA [20,21,22], and legume LAP1 [23], have been identified as involved in regulation of the anthocyanin biosynthetic pathway. In *Arabidopsis*, overexpression *AtPAP1* results in accumulation of anthocyanins [15].

In the present study, *Actinidia arguta* var. purpurea is characteristic kiwifruit species that produces oval shaped fruits containing a mauve outer exocarp and a purple-flesh endocarp, which is a key trait involving its unique regulation of anthocyanin metabolism. The transcriptome of *A. arguta* fruit sarcocarp was performed by Illumina sequencing. Furthermore, to identify candidate genes involved in anthocyanin biosynthesis, differentially expressed genes (DEGs) were analyzed during three fruit development stages. We identified the transcription factor AaMYB1 and confirmed its function in regulating anthocyanin biosynthesis. The transcriptomic datasets will facilitate *A. arguta* breeding and establish a strong foundation for studies of fruit development in *A. arguta*.

## 2. Results

### 2.1. Anthocyanin Pigment Measurement during Fruit Development

**Figure 1:**
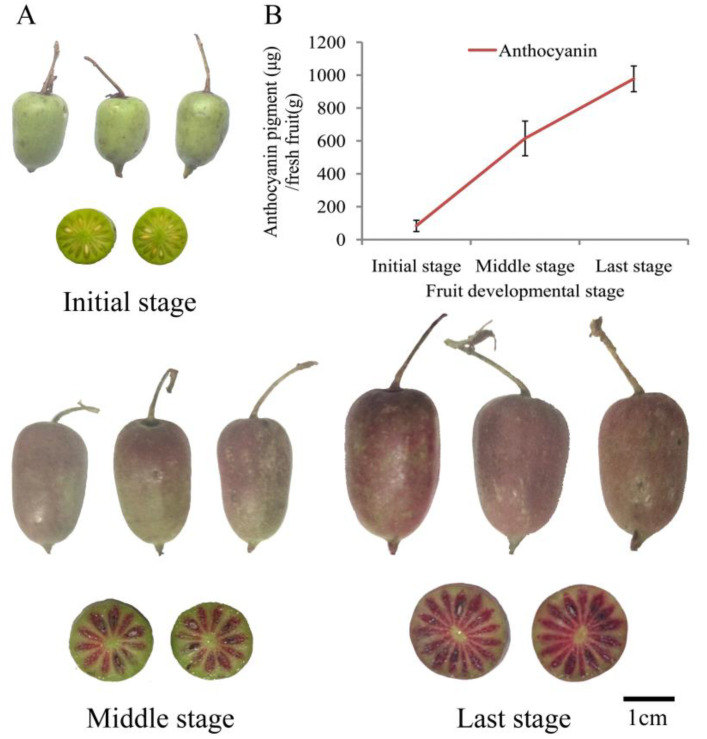
The anthocyanin pigment during fruit development of *A. arguta*. (**A**) Developmental stages of *A. arguta* fruit. (bar = 1 cm). (**B**) Anthocyanin pigment and concentration in fruit during three developmental stages.

Phenotypic differences were observed during the three stages of *A. arguta* fruit development. The most obvious phenotypic difference is the change of fruit color (Figure 1A). Thus, anthocyanin pigment was observed and its concentration was measured from the fruit sarcocarp of three developmental stages, including the initial stage, middle stage, and last stage. Generally, some fruit plants will accumulate anthocyanin in their fruits during the progression of fruit development. Very low levels of anthocyanin pigment were detected in fruit sarcocarp from the initial stage (green fruit). Anthocyanin pigment increased remarkably in the other two developmental stages. The analysis of fruit sarcocarp showed that anthocyanin pigment accumulated significantly as the fruit developed, which was consistent with visual observation (Figure 1B). 

### 2.2. RNA-Seq and De Novo Transcriptome Assembly

The RNA-Seq analyses were performed on *A. arguta* fruit sarcocarp during the three developmental stages. The total reads of each sample varied from 54.69 to 55.09 million (Table 1). The clean data of all samples were assembled from scratch by Trinity, and the assembly results were optimized and evaluated. The results showed that the de novo assembly produced 77,003 unigenes, and the average length of N50 was 1220 bp (Table S1). Benchmarking Universal Single-Copy Orthologs (BUSCOs) analysis determined the de novo transcriptome to be 80.1% complete with unigene, and 83.9% complete with transcript (Table S1). In general, the quality of the RNA-Seq dataset is considered appropriate for further analysis.

### 2.3. Functional Annotation and Enrichment Analysis

In our study, a total of 34.05% of the 77,003 assembled *A. arguta* unigenes were annotated to the UniProtKB/Swiss-Prot database, and 50.43% were annotated to the NCBI_NR database (Table S2 and Figure S1). Based on the NCBI_NR database, of the 38,836 unigenes with a similar alignment, the *A. arguta* unigenes were homologous to sequences in other species (Figure S2).

A total of 33,227 unigenes were annotated to the GO database, which can be divided into three categories: biological process, cellular component, and molecular function (Table S2). In the category of biological process, the GO terms of cellular process, metabolic process, biological regulation, localization, cellular component organization or biogenesis, response to stimulus, and developmental process enriched the most unigenes. In the category of cellular component, genes related to cell, membrane, organelle, and protein-containing complex were the most abundant. Binding, catalytic activity, transporter activity, structural molecule activity, and transcription regulator activity had the most unigenes in the category of molecular function (Figure S3). Moreover, many DEGs were also enriched in carbohydrate metabolism, amino acid metabolism, translation, folding, sorting and degradation, transport and catabolism, and signal transduction (Figure S4). 

### 2.4. Identification of Differently Expressed Genes (DEGs) Functional Categorization

**Figure 2:**
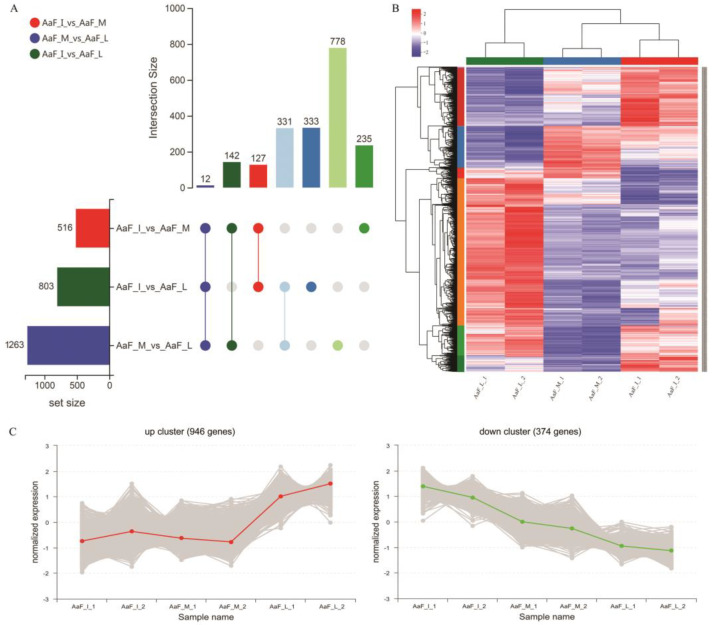
Analysis of transcriptome data from *A. arguta* fruit at three developmental stages. AaF_I, AaF_M, and AaF_L represent the samples at initial stage, middle stage, and last stage, respectively. (**A**) UpSetR plot of significantly differentially expressed genes. (**B**) Unigene cluster analysis between the three developmental stages. (**C**) Cluster trend analysis of DEGs between the three developmental stages.

To identify DEGs during *A. arguta* fruit development, we analyzed the transcriptome data at three developmental stages. Pairwise comparisons of samples from three developmental stages were performed with DESeq2 [24], and unigenes were considered to be differentially expressed using an adjusted *p*-value < 0.01 and an absolute log2 fold change ≥ 1. A total of 1958 unigenes were identified as differentially expressed between the three developmental stages (Table S3). In three paired comparisons of AaF_I vs. AaF_M, AaF_M vs AaF_L, and AaF_I vs AaF_L between the three developmental stages, the identified DEGs were 516, 1263, 803, respectively (Figure 2A).

Furthermore, metabolic pathways, such as hormonal regulation, pigmentation [25], sugar metabolism [26], and cell wall metabolism [27], are involved in synchronized regulation, which is also responsible for the complexity of fruit development. A cluster of genes with similar expression patterns tends to have functional relevance. In our study, some candidate genes were screened according to their correlation with fruit development. Through hierarchical clustering, 1,958 DEGs showed variations of gene expression in each stage in the heatmap (Figure 2B). Further, two main clusters, namely the up cluster and down cluster, were addressed by sub-cluster trend analysis (Figure 2C). The unigenes that were located within the same cluster had similar expression patterns during fruit development. The up cluster showed 946 DEGs were upregulated during the three developmental stages, whereas the down cluster showed 374 DEGs were downregulated. These data reveal that the developmental stages were key factors affecting related gene expression in all samples.

**Figure 3:**
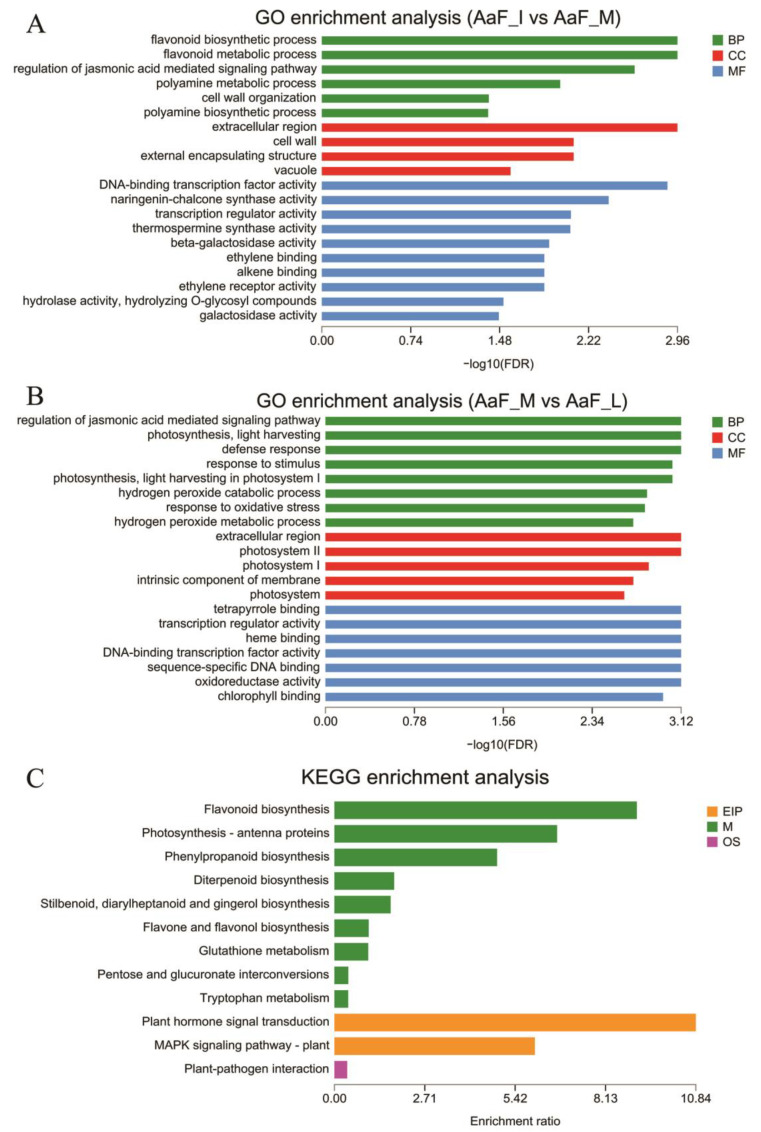
Functional classification of differentially expressed genes. (**A**) GO term enrichment analysis of DEGs between AaF_I and AaF_M. (**B**) GO term enrichment analysis of DEGs between AaF_M and AaF_L. (**C**) KEGG enrichment analysis of DEGs. Enrichment analysis used Fisher’s exact tests, and each bar represents a term or a pathway.

GO analysis was performed to clarify the functions of DEGs in comparisons between three developmental stages. GO can be divided into three categories: biological process, cellular component, and molecular function. In the comparison of AaF_I and AaF_M, the GO terms of flavonoid biosynthetic process, flavonoid metabolic process, regulation of jasmonic acid-mediated signaling pathway, and polyamine metabolic process enriched the most DEGs in the category of biological process. In the category of cellular component, the cell wall, extracellular region, and vacuole enriched the most DEGs. DNA-binding transcription factor activity and naringenin–chalcone synthase activity had the most DEGs in the category of molecular function (Figure 3A). Genes involved with anthocyanin metabolism were associated with the GO terms of flavonoid biosynthesis, vacuole, and transcription factor activity, all of which were significantly enriched, which is consistent with the visual changes of the fruit anthocyanin pigment phenotypes between AaF_I and AaF_M.

Furthermore, in the comparison of AaF_M and AaF_L, photosynthesis and response to stimulus enriched the most DEGs in the category of biological process. Meanwhile, extracellular region and photosystem enriched the most DEGs in the category of cellular component, and in the category of molecular function, such as binding, transcription regulator activity enriched the most DEGs (Figure 3B). To identify the active biological pathways in *A. arguta* fruit, KEGG pathways were used to analyze the pathway annotations of unigene sequences. There were also many DEGs participating in pathways related to anthocyanin synthesis (Figure 3C). The analysis found that the significant pathways involved in anthocyanin synthesis are phenylpropanoid biosynthesis (25 unigenes), flavonoid biosynthesis (20 unigenes), and anthocyanin biosynthesis (1 unigenes) (Table S4). These results suggest that intensive metabolic activities occur during fruit development and pigmentation in *A. arguta* fruit sarcocarp.

### 2.5. DEGs Involved in Anthocyanin Biosynthesis

**Figure 4:**
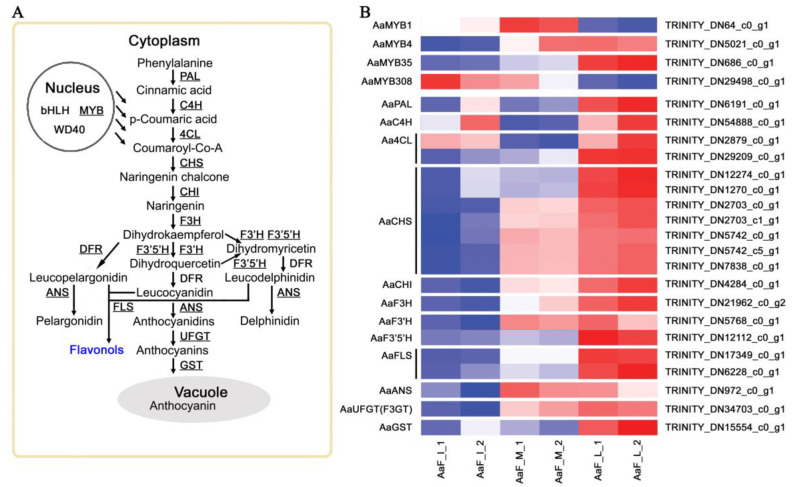
Expression patterns of the DEGs involved in anthocyanin biosynthesis at three developmental stages. (**A**) Schematic diagram of the anthocyanin biosynthetic pathway. DEGs are highlighted by underlines. (**B**) Heat maps of DEGs involved in anthocyanin biosynthesis at three developmental stages. Gene-expression level was measured using the TPM method. Horizontal axes represent three developmental stages, including initial stage (AaF_I_1 and AaF_I_2), middle stage (AaF_M_1 and AaF_M_2), last stage (AaF_L_1 and AaF_L_2), and vertical axes represent gene ID. Heat map shows the expression of genes ranging from blue (low expression) to red (high expression).

It was reported that the anthocyanin content of fruit is associated with the expression of structural and regulatory genes related to the anthocyanin biosynthesis pathway in apple [28,29], pear [30], and grape species [31]. Generally, anthocyanin biosynthesis is part of the flavonoid biosynthetic pathway. A total of 40 homologous DEGs relevant to the anthocyanin biosynthetic pathway were identified in *A. arguta* fruit development (Table S5). Additionally, more than one unique sequence encoding the same enzyme was found in the anthocyanin biosynthetic pathway. Most DEGs showed an expression pattern of significant upregulation from the initial or middle stage to the last stage (Figure 4). Meanwhile, DEGs homologous of the early genes, such as phenylalanine ammonia-lyase (*PAL*), cinnamate acid 4-hydroxylase (*C4H*), and 4-coumarate-CoA ligase (*4CL*), were downregulated from the initial stage to the middle stage, while few DEGs homologous of regulatory genes were downregulated from the middle stage to the last stage. Most genes encoding enzymes were upregulated to promote anthocyanin accumulation during the ripening process, which was consistent with the phenotype of fruit sarcocarp color.

In the comparison of AaF_I vs AaF_M, *AaPAL* (TRINITY_DN6191_c0_g1), *AaC4H* (TRINITY_DN54888_c0_g1), and *Aa4CL* (TRINITY_ DN2879_c0_g1) were downregulated, especially *AaC4H,* with its downregulation of more than 10-fold. DEGs homologous of chalcone synthase (CHS), chalcone–flavonone isomerase (CHI), Flavanone-3-hydroxylase (F3H), flavonoid 3′-hydroxylase (F3′H), flavonoid3′,5′-hydroxylase (F3′5′H), flavonol synthase (FLS), anthocyanidin synthase (ANS), and UDP-glucose: flavonoid-3-O-glycosyltransferase (UFGT) were upregulated, which supports the notion that anthocyanin synthesis is required for the change from green sarcocarp to purple sarcocarp. Compared AaF_L to AaF_M, DEGs homologous to PAL, C4H, 4CL, CHS, CHI, F3H, F3′H, F3′5′H, FLS, ANS, and UFGT were significantly upregulated, especially *AaCHS,* with its more than 16-fold upregulation, which also reflects a positive response to anthocyanin synthesis and accumulation. Generally, the initiation of anthocyanin biosynthesis begins with the CHS enzymatic catalysis that forms naringenin chalcone [32]. Next, CHI isomerizes naringenin chalcone to form a flavanone, naringenin [33]. Following the synthetic pathway, F3′H or F3′5′H further hydroxylates dihydrokaempferols to produce dihyroquercetin or dihyromyricetin [34]. Dihydroflavonol 4-reductase (DFR) catalyzes the reduction of dihydroflavonols (dihydrokaempferol, dihyroquercetin, and dihyromyricetin) to leucoanthocyanidins [35]. Subsequently, ANS converts leucoanthocyanidins into anthocyanidins, and UFGT stabilizes the unstable free hydroxyl group at the 3 position of the heterocyclic ring to form anthocyanins [36]. However, the UFGT gene homologue *AaF3GT* was upregulated more than 9-fold in the comparison of AaF_I vs. AaF_M, and only upregulated nearly 2-fold in the comparison of AaF_M vs. AaF_L, as its expression level was high. Some studies have also suggested that the UFGT is essential for fruit pigment precipitation in pears [37] and other fruits [38,39]. Furthermore, after anthocyanins are synthesized, they need to be transported to vacuoles. This process requires glutathione transferase (GST) proteins to physically bind to anthocyanins and act as anthocyanin carrier proteins [40]. *AaGST* (TRINITY_DN15554_c0_g1) was found to be significantly upregulated in the last phase, whereas its expression was restricted in the first two stages, which is consistent with the process law of anthocyanin pigment accumulation in fruit sarcocarp (Figure 4B).

### 2.6. Correlation Analysis of Genes Involved in Anthocyanin Biosynthesis

**Figure 5:**
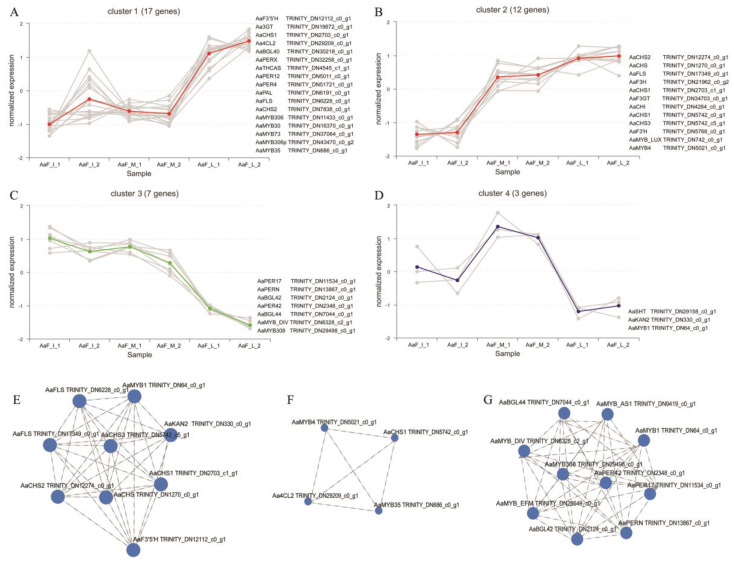
Expression pattern analysis of candidate genes involved with anthocyanin biosynthesis. (**A**–**D**) cluster trend analysis of candidate genes. Red line represents the overall upward trend, green line represents the overall trend of decline, blue line represents the trend rising first and then falling. (**E**–**G**) expression-correlation analysis of candidate genes by Spearman method.

Besides regulating gene expression in biosynthetic pathways, transcription factors also play a crucial role in secondary metabolism [41]. There were 133 transcription factors encoding unigenes among the DEGs identified through *A. arguta* fruit development. Most transcription factors were classified into the bHLH (13), ERF (27), MYB (19), and WRKY (17) families (Table S6). Previous studies indicated that transcription factors including MYB, bHLH, and WD40 proteins can form MBW protein complexes to regulate the expression of structural genes involved in the anthocyanin biosynthetic pathway [42]. Thus, to further analyze anthocyanin biosynthesis, we selected MYB transcription factor genes and key enzyme genes related to the flavonoid pathway, for expression-pattern cluster trend analysis and expression correlation analysis (Figure 5).

In this study, we found that 17 DEGs clustered into the cluster1 group were significantly upregulated between the middle and last stage of fruit development (Figure 5A), and 12 DEGs clustered into the cluster2 group were significantly upregulated between the initial and middle stage (Figure 5B). However, seven DEGs clustered into the cluster3 group were obviously downregulated during the three stages of fruit development (Figure 5C). Notably, three DEGs clustered into the cluster4 group were significantly upregulated between the initial and middle stage, then remarkably downregulated between the middle and last stage (Figure 5D).

Furthermore, we focused on MYB transcription factor DEGs including *AaMYB1* (TRINITY_DN64_c0_g1), *AaMYB308* (TRINITY_DN29498_c0_g1), *AaMYB4* (TRINITY_DN5021_c0_g1), and *AaMYB35* (TRINITY_DN686_c0_g1). In an expression correlation analysis, *AaMYB1* was shown to be significantly correlated with CHS, FLS, and F3’5’H genes (Figure 5E). *AaMYB4* and *AaMYB35* were interrelated, and correlated with CHS and 4CL genes (Figure 5F). *AaMYB308* was significantly correlated with Beta-glucosidase (BGL) and Peroxidase (PER) genes, while also correlated with other MYB transcription factor genes, such as *AaMYB1* (Figure 5G). However, BGL and PER were reported to be key enzymes in the lignin metabolic pathway of phenylpropanoid biosynthesis [43,44]. *AaMYB4* and *AaMYB35* have close homology to *AtMYB4* and *AtMYB35*, and AtMYB35 was reported to be involved in tapetum development [45], AtMYB4 was reported to function as a repressor of cinnamate 4-hydroxylase (C4H) to inhibit lignin biosynthesis [46]. Only *AaMYB1* has a near-homology to *MdMYB10* [22], *AtMYB75,* and *AtMYB113*, which is known to regulate required genes for flavonoid and anthocyanin biosynthesis [47]. Meanwhile, the expression of the *AaMYB1* transcription factor significantly increased from the initial to the middle stage of fruit development, which is an important phenotype process, with the sarcocarp beginning to appear purplish-red (Figure 5D).

### 2.7. Quantitative RT-PCR Verification and Correlation Analysis of Genes Involved in Anthocyanin Biosynthesis

Ten DEGs homologous of genes involved in anthocyanin biosynthesis were selected for qRT-PCR analysis (Figure S5). The results of qRT-PCR analysis were, in a great measure, consistent with those obtained from DEG analysis. There was a positive correlation between the expression of most candidate genes and fruit sarcocarp pigmentation.

### 2.8. Identification of AaMYB1 as a Regulator of Anthocyanin Biosynthesis

**Figure 6:**
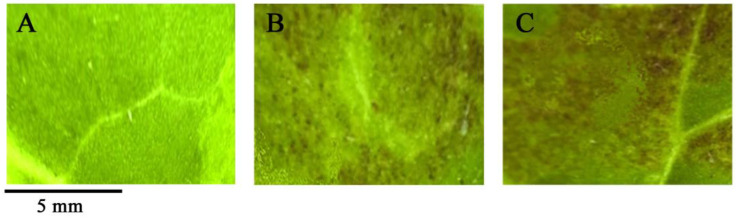
Transient transformation assays identify the activity of AaMYB1 for anthocyanin biosynthesis. (**A**) A representative *Nicotiana benthamiana* leaves image infiltrated with empty vector by transient transformation assays. (**B**) Patches of anthocyanin production in *Nicotiana benthamiana* leaves by AaMYB1. (**C**) Patches of anthocyanin production in *Nicotiana benthamiana* leaves by combination of AaMYB1 with AtTT8.

In apples, an MYB protein, MdMYB10, transformed into tobacco leaves together with MdbHLH3, can induce anthocyanin production, which implies that MYB is responsible for regulating anthocyanin biosynthesis [22]. In Arabidopsis, *AtMYB75* was also found to be co-expressed with *bHLH*s [47]. A candidate MYB transcription factor, AaMYB1, was selected to characterize the biological function of the MYBs in regulating anthocyanin biosynthesis in *A. arguta*. Induction of anthocyanin biosynthesis in transient assays was performed to study the function of AaMYB1 (Figure 6). When AaMYB1 was transformed together with AtbHLH42 (AtTT8) and co-expressed in leaves, anthocyanin patches were most apparent. Therefore, combining AtTT8 with AaMYB1 enhanced anthocyanin accumulation, which suggests that these two proteins may jointly regulate anthocyanin biosynthesis. The results of the transient assays suggest that AaMYB1 is a true regulator of anthocyanin biosynthesis in *A. arguta*.

## 3. Discussion

Fruit color is an important economic characteristic of fruit plants. The *A. arguta* de novo transcriptome of fruit sarcocarp development with annotation provides a new resource for studying fruit development. In our study, these data are important for the discovery of novel genes in the fruit ripening process of *A. arguta*, and may also facilitate the study of closely related species. Approximately 51% of the *A. arguta* unigenes were annotated to major databases such as the NR, Swiss-Prot, and KEGG databases (Table S2). However, non-coding regions or inadequate sequence length, or the lack of information in databases, may result in the approximately 49% of remaining unigenes have no annotations.

Through the pairwise comparison of samples from the three developmental stages, 1958 DEGs were identified in this study. Many DEGs were found to be involved in fruit development, and many key enzymes genes were identified to be related to anthocyanin biosynthesis, flavonoid biosynthesis, and phenylpropanoid biosynthesis. However, none of these candidate genes have been studied or reported in *A. arguta*. Thus, the biological function of these candidate genes will require more identification in future studies.

Generally, plants are subjected to various environmental stressors, and phytohormones play an important role in plant responses to these stressors [48]. It was reported that the expression of the anthocyanin biosynthesis-related regulators *PAP*, *PAP1,* and *GL3*, are induced by the activity of the jasmonic acid (JA)-signaling pathway in *Arabidopsis* [49]. Meanwhile, other studies have also suggested that JA mediates the biosynthesis and accumulation of anthocyanins in plants when they are subjected to adverse environmental stresses [50,51,52]. In our study, eight DEGs involved in regulation of the JA-mediated signaling pathway were downregulated from the initial stage to the middle stage of fruit development, and upregulated between the middle and last stages, supporting the idea that JA-signaling may participate in the anthocyanin accumulation process (Table S7). Furthermore, light as an environmental condition has always existed in the growth, development, and reproduction of plants [53], which seems to suggest that the JA-signaling may be simultaneously involved in the response to light. Meanwhile, plants always respond to changes in light intensity in the wild, which will also reflect alterations in plant color and represents one of the adaptive responses to light signals [54]. Our *A. arguta* samples, collected from the wild, also need to respond to changes in light intensity of their natural environments. Ten DEGs involved in photosynthesis were found, and nine of them were upregulated from the initial stage to the middle stage of fruit development, and downregulated between the middle and last stage (Table S8). Notably, most DEGs related to anthocyanin biosynthesis significantly increased during the fruit ripening process, from the middle stage to the last stage, which might reflect their ability to provide photoprotection. Although light is required for plants to generate energy for growth and development, there are potential risks in capturing light energy by the photosynthetic apparatus of plants [55]. In this regard, the accumulation of anthocyanins is a primary strategy to shield against excessive light energy through optical masking chloroplasts, as well as to avoid oxidative injury by directly scavenging excessive ROS, owing to the antioxidant properties of anthocyanins [56,57,58].

MYB-bHLH-WD repeat (MBW) complexes were considered to regulate anthocyanin biosynthetic pathways [42]. In our transcriptome analysis, AaMYB1 is the one of the significantly upregulated MYB genes between the initial and middle stages of fruit development, and is necessary and sufficient to activate anthocyanin biosynthesis (Figure 5D). The paralogs of AaMYB1, such as AtMYB75 in Arabidopsis [47], and MdMYB10 in apples [22], are regulators of anthocyanin biosynthesis in fruit. However, the AaMYB1 gene is downregulated during the middle and last stage of fruit development, which is related to the fruit ripening process (Figure S5). This may be because more regulatory factors are involved in the fruit ripening process, interfering with the expression of AaMYB1, or partially replacing its function. Additionally, the function of AaMYB1 is enhanced when co-expressed with AtTT8 in the transient transformation assays, which is consistent with previous reports using apples and pears [22]. These findings indicate that there may be some differences in the regulation of anthocyanin accumulation in *A. arguta* compared with other plants.

In addition, the expression-level changes of most candidate structural genes were significant in the anthocyanin biosynthetic pathway during the fruit development process. However, the expression level of the structural gene *AaANS* was high compared with other candidate structural genes in the initial stage of fruit development, and its expression level was upregulated in the middle and late stages compared with the initial stage. Meanwhile, the upregulation range of *AaANS* is smaller than that of other enzyme genes such as *AaCHS*. Furthermore, the expression of enzyme gene AaFLS was also upregulated, which formed some competition for AaANS catalytic substrates. Therefore, the interactions and relationships between candidate structural genes and transcription factors will need to be investigated further. In total, this study provides a valuable foundation for the understanding of sarcocarp anthocyanin biosynthesis in *A. arguta* fruit development and will facilitate *A. arguta* breeding.

## 4. Materials and Methods

### 4.1. Plant Materials

*Actinidia arguta* var. purpurea were located on the edge of a cliff on the Emei Mountain in China. The fruit samples were collected from the wild plants. The fruit samples were taken at 7 weeks, 12 weeks, and 16 weeks after flowering, which were divided into three stages including the initial stage, middle stage, and last stage, respectively. The sarcocarp samples were taken from the flesh of fruits without the skin or seed. The sarcocarp sample of each tube was collected from six pieces of flesh from six independent fruits, which were from three different chutes on three different vines. The fruits picked in the same stage were prepared into three tubes of independent sarcocarp samples as three independent replicates. The samples were prepared after the fruits were picked, then immediately placed in liquid nitrogen and stored at −80 °C for RNA extraction and further analysis. *Nicotiana benthamiana* with six leaves were used for genetic transformation, and grown in a growth chamber with a temperature setting of 22 °C and a light cycle of 16 h light/8 h dark.

### 4.2. Determination of Anthocyanin Content

Total anthocyanin was extracted from various fresh tissues using 1% HCl in methanol (*v*/*v*), and stored in the dark at 4 °C overnight with occasional shaking. The extracts were centrifuged at 10,000× *g* for 10 min at 4 °C, and the supernatant was taken out for determination of absorbance at 530 and 657 nm. Total anthocyanin content was quantified using the equation A_530_ − 0.25 × A_657_, which compensates for the contribution of chlorophyll and its degradation products with absorbance at 530 nm. Cyanidin3-glucoside was used as a reference standard. Three replicates were analyzed for each sample.

### 4.3. RNA Extraction, Library Preparation, and Sequencing

Total RNA was extracted from the sarcocarp tissue using TRIzol Reagent (Plant RNA Purification Reagent for plant tissue) according the manufacturer’s instructions (Invitrogen, Carlsbard, CA, USA), and genomic DNA was removed using DNase I (TaKara, Kusatsu, Japan). Then, the integrity and purity of the total RNA quality was determined by 2100 Bioanalyser (Agilent Technologies, Inc., Santa Clara, CA, USA) and quantified using the ND-2000 (NanoDrop Thermo Scientific, Wilmington, DE, USA). Only a high-quality RNA sample (OD260/280 = 1.8~2.2, OD260/230 ≥ 2.0, RIN ≥ 8.0, 28S:18S ≥ 1.0, >1 μg) was used to construct sequencing library. RNA purification, reverse transcription, library construction, and sequencing were performed at Shanghai Majorbio Bio-pharm Biotechnology Co., Ltd. (Shanghai, China) according to the manufacturer’s instructions (Illumina, San Diego, CA, USA). The RNA-seq transcriptome libraries were prepared using Illumina TruSeqTM RNA sample preparation Kit (San Diego, CA, USA). Poly(A) mRNA was purified from total RNA using oligo-dT-attached magnetic beads and then fragmented by fragmentation buffer. Taking these short fragments as templates, double-stranded cDNA was synthesized using a SuperScript double-stranded cDNA synthesis kit (Invitrogen, Carlsbard, CA, USA) with random hexamer primers (Illumina). Libraries were size-selected for cDNA target fragments on 2% Low Range Ultra Agarose, followed by PCR amplified using Phusion DNA polymerase (New England Biolabs, Boston, MA, USA). After being quantified by TBS380, the library preparations were sequenced on an Illumina Hiseq xten/NovaSeq 6000 platform (Illumina, San Diego, CA, USA).

### 4.4. De Novo Assembly and Annotation

The raw paired-end reads were trimmed and quality controlled by SeqPrep (https://github.com/jstjohn/SeqPrep, accessed on 2 November 2021) and Sickle (https://github.com/najoshi/sickle, accessed on 2 November 2021) with default parameters. Then, clean data from the samples were used for de novo assembly with Trinity (http://trinityrnaseq.sourceforge.net/, accessed on 2 November 2021) [59]. All of the assembled transcripts were searched against the NCBI protein nonredundant (NR), COG, and KEGG databases, using BLASTX to identify the proteins that had the highest sequence similarities with the given transcripts to retrieve their function annotations, and a typical cutoff of E-values less than 1.0 × 10^−5^ was set. BLAST2GO (http://www.blast2go.com/b2ghome, accessed on 2 November 2021) program [60] was used to obtain GO annotations of unique assembled transcripts for describing biological processes, molecular functions, and cellular components. Metabolic pathway analysis was performed using the Kyoto Encyclopedia of Genes and Genomes (KEGG, http://www.genome.jp/kegg/, accessed on 2 November 2021) [61]. We quantified the completeness of the de novo assembly by comparing the assembled transcript set against a set of highly conserved single-copy orthologs using BUSCO [62].

### 4.5. Differential Expression Analysis and Functional Enrichment

To identify differential expression genes (DEGs) between two different samples, the expression level of each transcript was calculated according to the transcripts per million reads (TPM) method. RSEM (http://deweylab.biostat.wisc.edu/rsem/, accessed on 2 November 2021) [63] was used to quantify gene abundances. Essentially, differential expression analysis was performed using the DESeq2 [24] with Q value ≤ 0.05, and DEGs with |log2FC| > 1 and Q value ≤ 0.01 (DESeq2 or EdgeR) were considered to be significantly differently expressed genes. In addition, functional-enrichment analyses, including GO and KEGG, were performed to identify which DEGs were significantly enriched in GO terms and metabolic pathways at Bonferroni-corrected *p*-value ≤ 0.05 compared with the whole-transcriptome background. GO functional enrichment and KEGG pathway analysis were carried out by Goatools (https://github.com/tanghaibao/Goatools, accessed on 2 November 2021) and KOBAS (http://kobas.cbi.pku.edu.cn/home.do, accessed on 2 November 2021) [64]. Hierarchical clustering of DEGs was performed in R using Z-scaled TPM data and clustering based on Pearson’s correlation and complete linkage clustering. Z-scaled TPM values were grouped by k-means clustering, clusters were chosen based on the least within-group sum of squares method. Correlation analyses of DEGs were calculated based on Spearman’s correlation coefficients.

### 4.6. Quantitative RT-PCR Analysis

To test the expression results from the RNA-seq analysis, we determined the expression levels of the selected genes by the q-PCR method with designed primers (Table S9), and 2 × SYBR Green RT-PCR Master Mix (Roche, Basel, Switzerland) was used as a fluorescent reporter. The PCR reaction program was set according to the manufacturer’s instructions (Roche). The *A. arguta* reference gene (β-Actin) was used for normalization. The relative gene expression was calculated using the 2^−ΔΔc(t)^ method.

### 4.7. Transient Transformation of Nicotiana Benthamiana Leaves

Transient transformation of tobacco assays was mainly performed with reference to those previously reported [22]. Agrobacterium cultures were incubated in LB medium at 28 °C overnight. Cells were collected by centrifugation at 3000× *g* for 10 min and resuspended in infiltration buffer (half-strength MS medium supplemented with 2% sucrose and 200 μM acetosyringone and pH was adjusted to 5.6) with a 0.2 of OD600 value. The coding regions of AaMYB1 and AtTT8, were cloned into pGreen II 62-SK transient expression vectors to generate constructs, using primers listed in Table S9, respectively. Agrobacterium with constructs of empty control, *AaMYB1*, and *AaMYB1* combined with *AtTT8* were infiltrated into *N. benthamiana* leaves and observed for pigmentation after 4 days. Digital photographs were taken 6 days after infiltration.

## Figures and Tables

**Table 1:** Summary of sequence assembly after sequencing.

Sample	Raw Reads	Clean Reads	Mapped Reads	Q20 (%)	Q30 (%)	GC Content (%)	Mapped Ratio to Assembling
AaF_I_1	55,090,506	55,082,814	46,864,370	100	100	46.42	85.08%
AaF_I_2	54,921,752	54,912,276	46,617,616	100	100	46.34	84.89%
AaF_M_1	54,853,482	54,844,046	46,723,014	100	100	46.59	85.19%
AaF_M_2	55,063,856	55,056,096	46,740,038	100	100	46.46	84.90%
AaF_L_1	54,691,116	54,680,852	46,323,952	100	100	46.52	84.72%
AaF_L_2	54,776,194	54,767,628	45,765,756	100	100	46.21	83.56%

## Supplementary Materials

The following supporting information can be downloaded at: https://www.mdpi.com/article/10.3390/ijms232012120/s1.
