# Supplementary material for: De Novo Assembly of a Sarcocarp Transcriptome Set Identifies AaMYB1 as a Regulator of Anthocyanin Biosynthesis in Actinidia arguta var. purpurea
Source: Int J Mol Sci. 2022 Oct 11;23(20):12120. doi: 10.3390/ijms232012120 (PMC9603036; doi:10.3390/ijms232012120)
Supplement: Supplementary file 1 [file ijms-23-12120-s001.zip › Supplementary Figures.pdf]

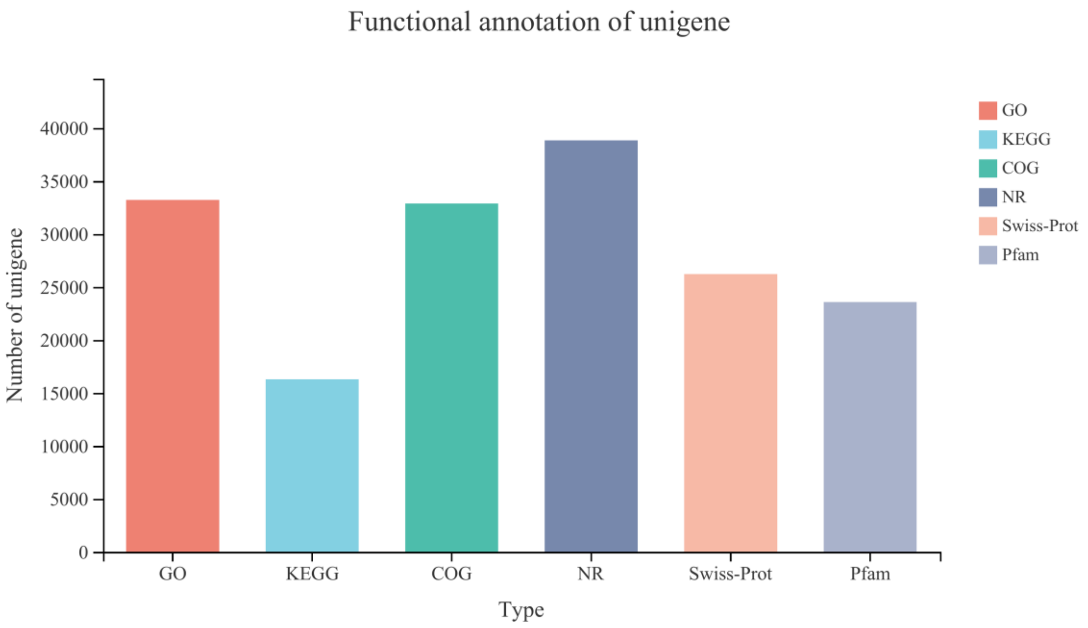

Figure S1. Functional annotation of unigene

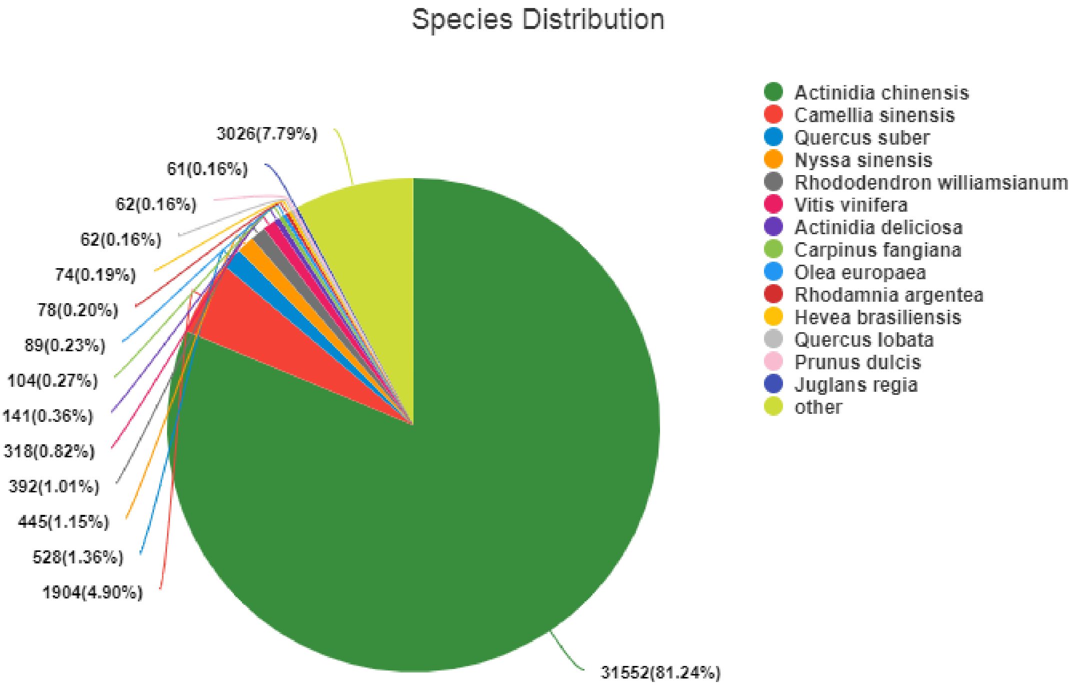

Figure S2. Species distribution

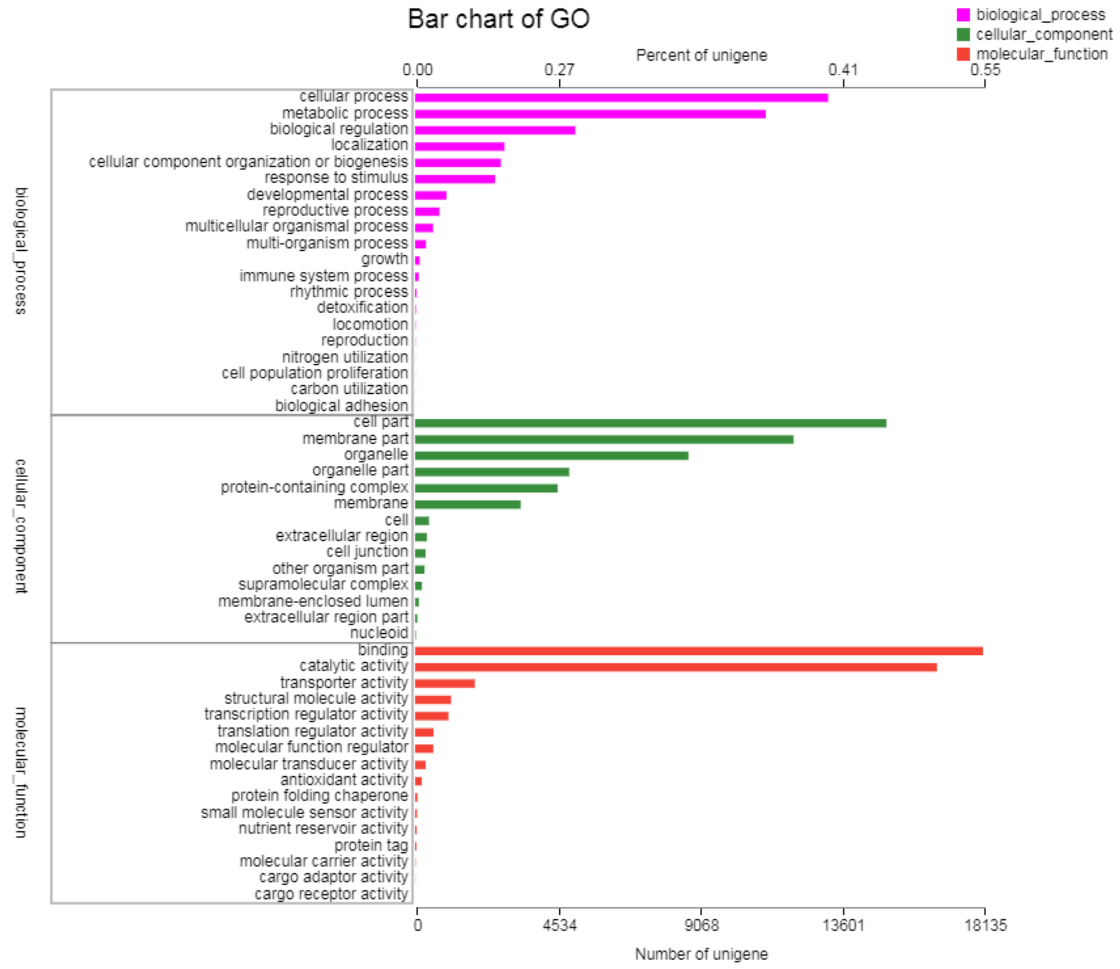

Figure S3. GO classification

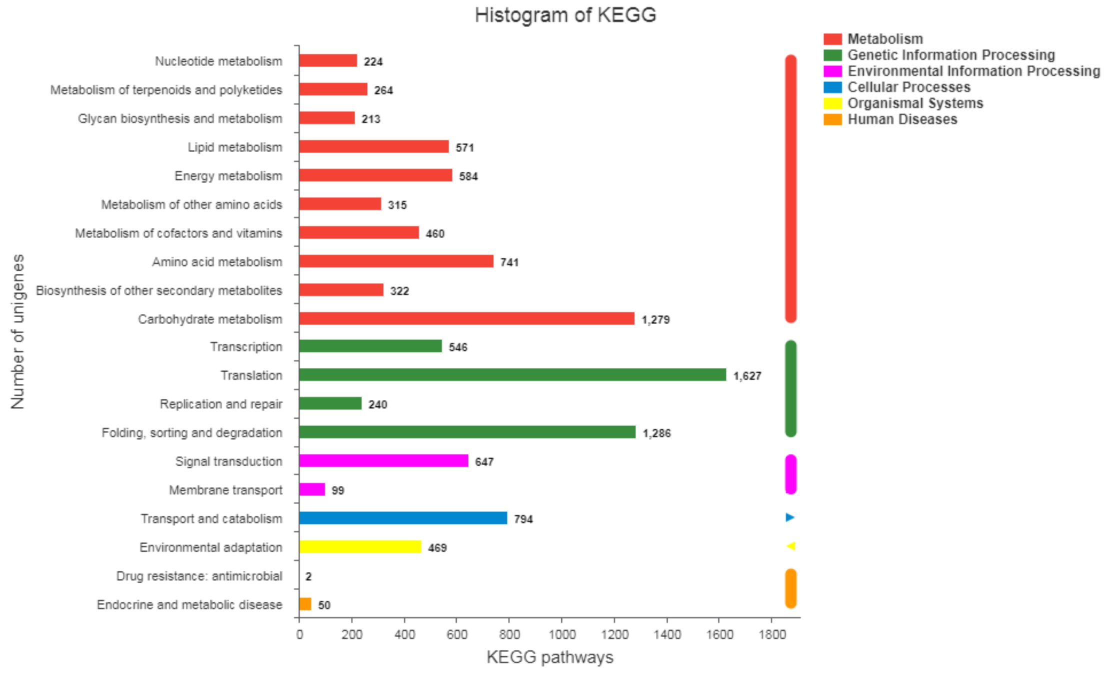

Figure S4. Keeg statistics

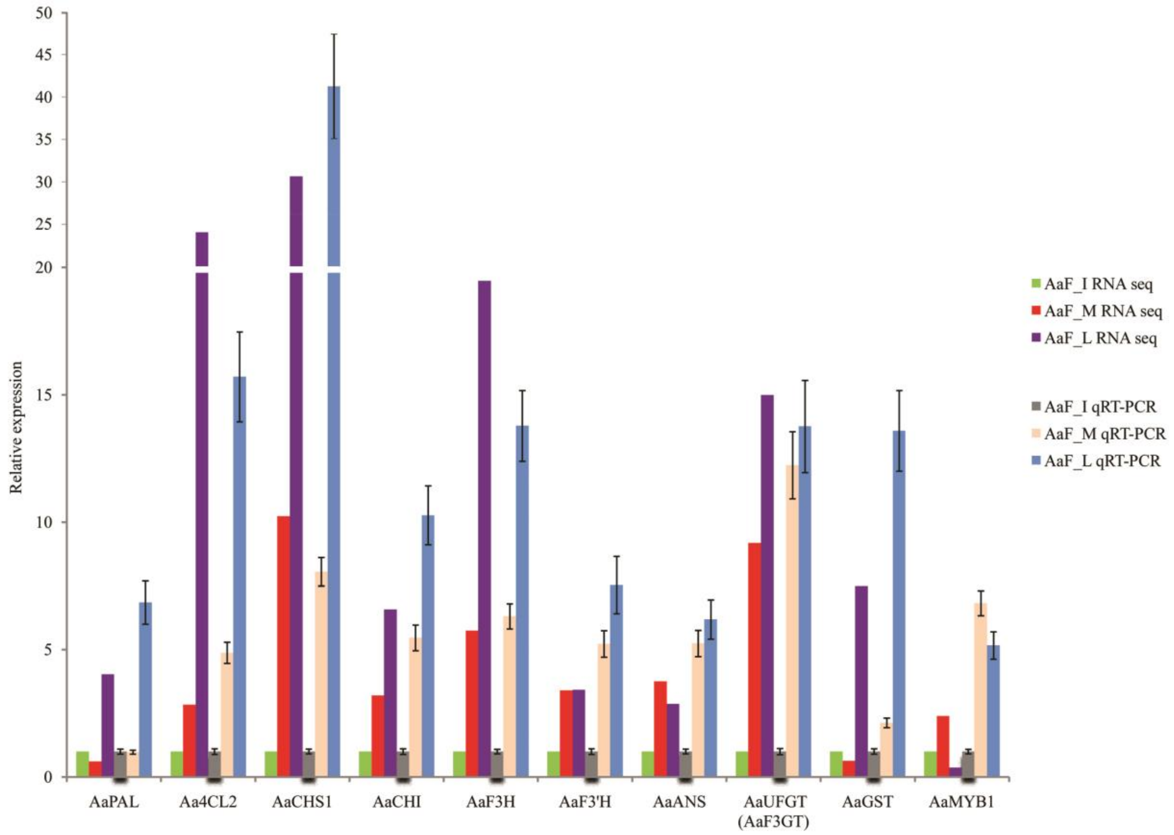

Figure S5. Expression levels of the selected genes from RNA sequencing data and qRT-PCR at three developmental stages including initial stage (AaF_I), middle stage (AaF_M), and last stage (AaF_L). Expression values were displayed by the fold of the expression at initial stage.
